# Psychosocial distress and quality of life in patients after radical cystectomy – one year follow-up in 842 German patients

**DOI:** 10.1007/s11764-023-01400-6

**Published:** 2023-05-10

**Authors:** Henning Bahlburg, Tabea Hellmann, Karl Tully, Marius Cristian Butea-Bocu, Moritz Reike, Florian Roghmann, Joachim Noldus, Guido Müller

**Affiliations:** 1 Department of Urology, Marien Hospital Herne, Ruhr-University Bochum, Hölkeskampring 40, 44625 Herne, Germany; 2 Center for Urological Rehabilitation, Kliniken Hartenstein, Bad Wildungen, Germany

**Keywords:** Bladder cancer, Radical cystectomy, Psychosocial distress, Quality of life, Urinary diversion

## Abstract

**Purpose:**

This study aims to report on psychosocial distress and QoL in bladder cancer patients after radical cystectomy (RC) and urinary diversion to obtain a better basis for patient counseling and postoperative care.

**Methods:**

The study relied on prospectively collected data for 842 patients, who underwent three weeks of inpatient rehabilitation after RC and creation of an ileal conduit (IC) or ileal neobladder (INB). Data on QoL and psychosocial distress were collected by validated questionnaires. Multivariate logistic regression was performed to identify predictors for high psychosocial distress.

**Results:**

Four-hundred and forty-seven patients (326 male, 121 female) received an IC, while 395 patients (357 male, 38 female) received an INB. Health-related QoL improved steadily in the whole cohort during follow-up. Patients with an INB reported better physical function but suffered more from diarrhea and financial worries. Patients with an IC reported reduced satisfaction with their body image, increased worries about the future, and suffered more from constipation. Psychosocial distress increased significantly during follow-up. One year after surgery, 43.1% of patients suffered from high psychosocial distress. Multivariate regression analysis identified age ≤ 59 years (OR 1.731; CI 1.056–2.838; *p* = 0.030) and lymph node metastases (OR 2.073; CI 1.133–3.793; *p* = 0.018) as independent predictors for high psychosocial distress.

**Conclusion:**

QoL improves significantly in all patients one year after RC. However, psychosocial distress remains high in a substantial number of patients.

**Implications for Cancer Survivors:**

To prevent chronic psychological disorders, easily accessible opportunities for psycho-oncological counseling are needed for patients following RC.

**Supplementary Information:**

The online version contains supplementary material available at 10.1007/s11764-023-01400-6.

## Introduction

With advanced screening methods and improved health care, the rate of cancer survivors will increase [1]. But fatigue, pain, anxiety, depression, diminished self-esteem, and impaired cognitive function may persist in these patients [2-4]. It is estimated that between 30 and 75% of cancer patients develop depression, anxiety, or an adaptive disorder during therapy [5-8]. That is why high psychosocial distress has been discussed as a “sixth vital sign” in tumor patients [9]. Overall, the greatest psychosocial distress appears to be caused by fear of disease progression [10]. In breast cancer, low psychosocial distress has been identified to predict longer recurrence-free and overall survival [11]. Psycho-oncological support should be considered a standard component of cancer treatment, including screening, monitoring, and treatment of psychosocial distress [12].

Radical cystectomy (RC) has seen an increase of 28% in cases between 2006 and 2019 in Germany. Ileal conduit (IC) and ileal neobladder (INB) are the most common types of urinary diversion [13, 14]. Five-year survival after RC is at about 60%, with a range of 25% to 72% depending on tumor stage [15]. Up to 35% of patients show clinical symptoms of depression after RC [16]. Even before surgery, up to 45% of patients suffer from high psychosocial distress. Advanced tumor stage was associated with high distress, while gender, age, and type of urinary diversion were not identified as predictors of high psychosocial distress in patients with bladder cancer [17, 18].

Quality of life (QoL) after RC is often reduced, e.g., by pain or sleeplessness [19]. Patients with an INB report better general state of health, global QoL, and physical function than patients with an IC [19-21]. However, other authors argue that there is no difference in QoL between patients with an IC and an INB [22-26]. Overall, RC leads to a deprived body image, but patients with an INB are often more content with their choice of urinary diversion [27, 28]. Health-related QoL has been identified to predict overall survival in several tumor entities [29-33].

In order to reach the important goal of reintegration into daily life, German social laws entitle cancer patients to an average of three weeks of inpatient rehabilitation (IR). The guideline of the German Society of Urology recommends that all patients be offered several weeks of IR after RC for bladder cancer [34]. It is assumed that in Germany the majority of patients after RC and urinary diversion participate in IR as recommended. Therefore, the design of our study allows us to report on QoL and psychosocial distress from a large number of patients within a recent period.

## Methods

This prospective study is based on clinical data of patients with urothelial carcinoma of the bladder who had received RC and IC or INB creation in various hospitals across Germany and who were treated in a specialized center for urological IR (Kliniken Hartenstein, Bad Wildungen, Germany) between April 2018 and December 2019. The study protocol was approved by an institutional research committee (research authorization number FF30/2017). Validated and established questionnaires were used to assess QoL and psychosocial distress at the end of IR (T1), and both 6 (T2) and 12 months (T3) post-surgery. Patients provided information regarding their education, occupation, and income at the beginning of IR. Based on these factors, the patients’ socioeconomic status was calculated [35].

### Quality of life questionnaire—EORTC QLQ-C30

The EORTC QLQ-C30, issued by the European Organization for Research and Treatment of Cancer (EORTC) is a questionnaire specially designed to evaluate QoL in cancer patients [1, 36]. It consists of 30 scored items and includes symptom scales such as dyspnea, loss of appetite, constipation, and diarrhea as well as functional scales such as physical function, cognitive function, emotional function, and social function. A high score in the functional scales equates to a high QoL, while a high score in the symptom scales mirrors a higher burden of symptoms.

### Quality of life questionnaire – EORTC QLQ-BLM30

The QLQ-BLM30 is an addendum to the QLQ-C30 and was developed to evaluate QoL after RC. Disease-specific items such as micturition problems or problems concerning maintenance of the urostomy as well as concerns regarding the future or a deprived body image are assessed [37]. Results are interpreted following the QLQ-C30.

### Questionnaire on stress in cancer patients – QSC-R10

Patients were screened for psychosocial distress using the Questionnaire on Stress in Cancer Patients (QSC-R10), a standardized and validated 10-item self-assessment instrument [38]. The 10 items cover the most relevant psychosocial aspects of everyday life in cancer patients. Symptoms such as pain, fatigue, and weakness as well as fear of disease progression, sleep disorders, or lack of information regarding the disease and its treatment are assessed. The 10 items are answered on a scale of 0 (“not applicable”) to 5 (“very high burden”). The QSC-R10 total score is calculated by adding up the single items. A sum ≥ 15 mirrors a high psychosocial burden and should trigger psycho-oncological counseling.

### Statistical analysis

Descriptive statistics of categorical variables comprised frequencies and proportions, whereas medians and interquartile ranges were presented for continuous variables. Between-group comparisons were analyzed using the Mann–Whitney U test or Chi-square test (Pearson) as appropriate. The Wilcoxon test was used to compare changes in quantitative variables, while the Chi-square test (McNemar) was used to compare changes in proportions. Multivariate logistic regression was performed to identify predictors for high distress. Significance was considered at *p* < 0.05. Analyses were performed by using IBM SPSS version 29.

## Results

A total of 842 patients (683 male, 159 female) from 135 different hospitals in Germany were included in this study. IR started at a median of 28 days (IQR 23–34) and ended with a median of 50 days (IQR 47–58) after surgery. The response rates for the follow-up survey were 80.5% (*n* = 678) at T2 and 68.2% (*n* = 574) at T3.

Four-hundred and forty-seven patients (326 male, 121 female) received an IC, while 395 (357 male, 38 female) received an INB. Female patients received an IC significantly more often than an INB (ratio 3:1; *p* < 0.001). Patients receiving an IC were significantly older (median 73 vs 64 years) and more often suffered from cardiovascular diseases (72.9% vs 55.9%; *p* < 0.001) and diabetes mellitus (16.1% vs 10.6%; *p* < 0.021). An advanced tumor stage (≥ pT3a) was significantly more often present in patients with an IC than in patients with an INB (41.4% vs 24.1%; *p* < 0.001). Consequently, lymph node metastases were also significantly more often found in patients with an IC (19.9% vs 11.8%; *p* < 0.001). Within the first 6 months after RC, 109 patients (16.1%) underwent chemotherapy, while an additional 22 patients (3.8%) received chemotherapy in the months 7–12 after RC. Further patients characteristics can be found in Table 1.

**Table 1:** Baseline characteristics of 842 patients after radical cystectomy for bladder cancer

Variable	Total	Conduit	Neobladder	p*
Patients, *n* (%)	842 (100.0)	447 (53.1)	395 (46.9)	
Age (years), Median (IQR)	68 (62–75)	73 (67–78)	64 (58–69)	< 0.001
≤ 59 years, *n* (%)	150 (17.8)	27 (6.0)	123 (31.1)	< 0.001
60–69 years, *n* (%)	307 (36.5)	125 (28.0)	182 (46.1)	< 0.001
≥ 70 years, *n* (%)	385 (45.7)	295 (66.0)	90 (22.8)	< 0.001
Gender, *n* (%)				
Male	683 (81.1)	326 (72.9)	357 (90.4)	< 0.001
Female	159 (18.9)	121 (27.1)	38 (9.6)	< 0.001
Karnofsky performance status (%), Median (IQR)	80 (70–80)	70 (70–80)	80 (70–80)	0.021
BMI (kg/m^2^), Median (IQR)	25 (23–28)	25 (23–28)	25 (23–27)	0.211
< 30, *n* (%)	743 (88.2)	390 (87.2)	353 (89.4)	0.341
≥ 30, *n* (%)	99 (11.8)	57 (12.8)	42 (11.6)	0.341
Cardiovascular disease, *n* (%)	547 (65.0)	326 (72.9)	221 (55.9)	< 0.001
Diabetes, *n* (%)	114 (13.5)	72 (16.1)	42 (10.6)	0.021
Socio-economic status**				
Lower social class	399 (54.1)	232 (61.1)	167 (46.6)	< 0.001
Middle social class	265 (35.9)	123 (32.4)	142 (39.7)	0.039
Upper social class	74 (10.0)	25 (6.6)	49 (13.7)	0.001
Working patients	230 (27.3)	51 (11.4)	179 (45.3)	< 0.001
Neoadjuvant chemotherapy, *n* (%)	83 (9.9)	37 (8.3)	46 (11.6)	0.102
Method of surgery, *n* (%)				
Robot-assisted cystectomy	93 (11.0)	46 (10.3)	47 (11.9)	0.458
Open cystectomy	749 (89.0)	401 (89.7)	348 (88.1)	0.458
Tumor stage, *n* (%)				
≤ pT2	562 (66.7)	262 (58.6)	300 (75.9)	< 0.001
≥ pT3	280 (33.3)	185 (41.4)	95 (24.1)	< 0.001
Lymph node-positive, n (%)***	131 (16.1)	86 (19.9)	45 (11.8)	0.002
No. of lymph nodes removed, Median (IQR)	17 (12–25)	17 (12–26)	17 (12–24)	0.765

*Abbreviations*: *IQR* interquartile range, *BMI* Body mass index, *CCI* Charlson comorbidity index

*Mann–Whitney-U test or Chi-square test (Pearson) as appropriate

**data available for 738 patients (conduit *n* = 380 and neobladder *n* = 358)

***data available for 815 patients (conduit *n* = 433 and neobladder *n* = 382)

Outcome parameters for QoL and psychosocial distress are shown in Supplement 1 (QLQ-C30) and Tables 2 (QLQ-BLM30) and 3 (QSC-R10).

**Table 2:** QLQ-BLM30 domains after radical cystectomy – conduit versus neobladder

Variable	Total mean (SD)	Conduit mean (SD)	Neobladder mean (SD)	p*
Urinary symptoms				
T1			45.3 (19.7)	
p**			< 0.001	
T2			38.0 (20.2)	
p***			< 0.001	
T3			34.1 (20,7)	
Urostomy problems				
T1		27.9 (21.3)		
p**		0.017		
T2		25.1 (21.1)		
p***		0.449		
T3		24.9 (21.5)		
Future perspective				
T1	45.4 (30.9)	47.7 (31.6)	42.8 (29.9)	0.043
p**	< 0.001	0.001	0.006	
T2	49.2 (31.0)	51.3 (31.2)	47.0 (30.7)	0.088
p***	< 0.001	0.033	0.006	
T3	45.8 (32.1)	47.7 (32.3)	43.9 (31.8)	0.179
Abdominal bloating / flatulence				
T1	28.9 (26.1)	31.3 (26.2)	26.3 (25.8)	0.003
p**	< 0.001	0.210	< 0.001	
T2	32.8 (27.8)	33.6 (27.7)	31.9 (27.9)	0.361
p***	0.269	0.562	0.303	
T3	31.9 (27.1)	32.2 (27.4)	31.6 (26.8)	0.838
Self-esteem / body image				
T1	30.9 (29.9)	32.1 (31.3)	29.4 (28.3)	0.498
p**	< 0.001	< 0.001	< 0.001	
T2	38.5 (31.2)	38.2 (31.9)	38.8 (30.5)	0.695
p***	0.009	0.151	0.025	
T3	36.1 (29.9)	35.5 (30.8)	36.6 (28.9)	0.458

*Abbreviations: T1* end of inpatient rehabilitation, *T2* 6 months after discharge from inpatient rehabilitation, *T3* 12 months after discharge from inpatient rehabilitation, *SD* standard deviation

*Mann–Whitney-U test

**Wilcoxon-test (T1 vs. T2)

***Wilcoxon-test (T2 vs. T3)

**Table 3:** Psychosocial distress (QSC-R10) after inpatient rehabilitation following radical cystectomy – conduit versus neobladder

Variable	Total	Conduit	Neobladder	p*
Total score				
T1, Median (IQR)	10 (4–18)	11 (5–18)	8 (4–17)	0.008
p**	< 0.001	< 0.001	< 0.001	
T2, Median (IQR)	13 (7–22)	14 (7–23)	12 (6–21)	0.292
p***	0.296	0.976	0.117	
T3, Median (IQR)	13 (6–21)	13 (7–22)	12 (5–21)	0.158
Cut-off ≥ 15				
T1, *n* (%)	290 (34.9)	168 (38.2)	122 (31.2)	0.035
p**	< 0.001	< 0.001	< 0.001	
T2, *n* (%)	253 (43.8)	132 (45.8)	121 (41.7)	0.319
p***	0.236	0.280	0.651	
T3, *n* (%)	215 (43.1)	109 (44.9)	106 (41.4)	0.437

*Abbreviations*: *QSC-R10* questionnaire on stress in cancer patients (10 items), *T1* end of inpatient rehabilitation (data available for 831 patients (conduit n = 440 and neobladder *n* = 391)), *T2* 6 months after discharge from inpatient rehabilitation (data available for 578 patients (conduit *n* = 288 and neobladder *n* = 290)), *T3* 12 months after discharge from inpatient rehabilitation (data available for 499 patients (conduit *n* = 243 and neobladder *n* = 256)), *IQR* interquartile range

*Mann–Whitney-U test, or Chi-square test (Pearson) as appropriate

**Wilcoxon-test or Chi-square test (McNemar) as appropriate (T1 vs. T2)

***Wilcoxon-test or Chi-square test (McNemar) as appropriate (T2 vs. T3)

## QoL and psychosocial distress at the end of IR (T1)

Health-related QoL, cognitive function, social function, and role function did not differ significantly between IC and INB patients at T1. However, patients with an INB performed better concerning emotional function (72.8 vs 67.4; *p* = 0.006) and physical function (68.0 vs 61.0; *p* < 0.001). Accordingly, patients with an IC were more worried about health-related developments (47.7 vs 42.8 *p* = 0.043) and suffered more from gastrointestinal symptoms (31.3 vs 26.3; *p* = 0.003). Financial problems were more common in patients with an INB compared to patients with an IC (24.5 vs 17.3; *p* = 0.001).

Median psychosocial distress at T1 was lower in patients with an INB than in patients with an IC (8 points (IQR 4–17) vs 11 points (IQR 5–18); *p* = 0.008). Overall, 34.9% of all patients reported high psychosocial distress (QSC-R10 ≥ 15) at T1. Patients with an INB were significantly less likely to be impacted by high psychosocial distress than patients with an IC (31.2% vs 38.2%; *p* = 0.035).

## QoL and psychosocial distress 6 months post-surgery (T2)

The mean value of global QoL improved significantly from 55.8 at T1 to 62.9 at T2 (*p* < 0.001). The improvements were observed in patients with an IC (61.9 vs 55.6; *p* < 0.001) as well as in patients with an INB (64.0 vs 56.0; *p* < 0.001) and did not differ significantly between the cohorts (*p* = 0.292). Both physical function (73.5 vs 64.3) and role function (58.1 vs 48.5) improved significantly in the whole cohort (*p* < 0.001). Patients with an INB reported significantly better physical function when compared to patients with an IC (78.4 vs 68.9; *p* < 0.001). However, emotional function worsened slightly in all patients (66.1 vs 70.0), while cognitive function and social function worsened only in patients with an IC. Fatigue regressed significantly at T2 in all patients (45.2 vs 40.7; *p* = 0.002), especially in patients with an IC. Meanwhile, financial problems (27.1 vs 24.5) and dyspnea (27.9 vs 20.2) increased in patients with an INB. Financial problems were still more common in patients with an INB compared to patients with an IC (27.1 vs 19.1; *p* = 0.002). Constipation regressed in the whole cohort (15.8 vs 19.8; *p* = 0.044), while diarrhea increased (27.2 vs 18.2; *p* < 0.001). Patients with an INB suffered less from constipation but reported diarrhea significantly more often than patients with an IC. Micturition problems decreased significantly in patients with an INB (38.0 vs 45.3; *p* < 0.001). However, future worries (47.0 vs 42.8; *p* = 0.006), flatulence (31.9 vs 26.3; *p* < 0.001), and body image (38.8 vs 29.4; *p* < 0.001) worsened significantly. In patients with an IC, problems with the urostomy decreased significantly (25.1 vs 27.9; *p* = 0.017) while future worries (51.3 vs 47.7; *p* = 0.001) and body image (38.2 vs 32.1; *p* < 0.001) worsened significantly.

Psychosocial distress increased significantly from 10 (IQR 4–18) at T1 to 13 points (IQR 7–22) at T2 (*p* < 0.001) in all patients. Meanwhile, an increasing number of patients reported high psychosocial distress (34.9% at T1 vs 43.8% at T2; *p* < 0.001). Again, at T2, no significant differences between the cohorts were observed (INB 45.8.% vs. IC 41.7%; *p* = 0.319).

## QoL and psychosocial distress 12 months post-surgery (T3)

In patients with an INB, QoL (65.8 vs 64.0; *p* = 0.012), physical function (80.5 vs 78.4; *p* = 0.004), role function (66.1 vs 60.7; *p* = 0.007), emotional function (69.0 vs 66.7; *p* = 0.003 and social function (64.2 vs 60.9; *p* = 0.001) improved significantly from T2 to T3. Patients with an IC, however, did not report any significant improvements in global QoL or functional subscales. HRQoL at T3 was significantly worse in patients undergoing adjuvant chemotherapy than in patients not undergoing treatment (54.7 vs 66.6; *p* < 0.001). Dyspnea in patients with an IC increased significantly (32.1 vs. 34.2; *p* = 0.011). In comparison to patients with an IC, those with an INB reported superior physical function (80.5 vs 69.2; *p* < 0.001) and role function (66.1 vs 57.3; *p* < 0.001), and significantly less fatigue (35.8 vs 41.9; *p* = 0.010), dyspnea (27.1 vs 34.2; *p* = 0.012), sleeplessness (31.6 vs 38.3; *p* = 0.016 and loss of appetite (11.2 vs 16.9; p = 0.005). Also, at T3, patients with an INB were significantly less troubled by constipation (16.8 vs 26.7; *p* < 0.001) but suffered more from diarrhea (28.5 vs 13.5; *p* < 0.001). Furthermore, financial worries were more common in patients with an INB (23.6 vs 15.9; *p* = 0.006). Future worries decreased significantly in the whole cohort from 49.2 at T2 to 45.8 at T3 (*p* < 0.001). These improvements were observed in patients with an IC as well as in patients with an INB. Additionally, patients with an INB reported fewer micturition problems (34.1 vs 38.0; *p* < 0.001) and fewer problems concerning their body image (36.6 vs 38.8; *p* = 0.025).

Twelve months after RC, high psychosocial distress (QSC-R10 ≥ 15) persisted in 43.1% of all patients with no significant differences between patients with IC and an INB. Univariate regression analysis identified lymph node metastases as a predictor for high psychosocial distress (OR 2.123; CI 1.194–3.777; *p* = 0.010). Multivariate regression analysis identified age ≤ 59 years (OR 1.731; CI 1.056–2.838; *p* = 0.030) and lymph node metastases (OR 2.073; CI 1.133–3.793; *p* = 0.018) as independent predictors for high psychosocial distress (Table 4).

**Table 4:** Regression analysis to identify predictors of high distress (QSC-R10 score ≥ 15) one year after discharge from inpatient rehabilitation following radical cystectomy

Variable	Univariate		Multivariate	
	OR (95% CI)	*p*	OR (95% CI)	*p*
Neobladder vs. Conduit	0.869 (0.609–1.239)	0.437	0.773 (0.522–1.145)	0.199
male vs. female	1.011 (0.619–1.652)	0.965	1.073 (640–1.800)	0.790
Age ≤ 59 years vs. ≥ 60 years	1.568 (0.992–2.479)	0.054	1.731 (1.056–2.838)	0.030
Tumor stage ≥ pT3 (yes vs. no)	1.235 (0.817–1.869)	0.316	1.085 (0.692–1.699)	0.723
Positive nodal stage (yes vs. no)	2.123 (1.194–3.777)	0.010	2.073 (1.133–3.793)	0.018

*Abbreviations*: *QSC-R10* questionnaire on stress in cancer patients (10 items), *OR* odds ratio, *CI* confidence interval

## Discussion

Maintaining or restoring QoL is an important goal in the treatment of cancer. Measuring QoL makes it possible to evaluate the success of a treatment and patient satisfaction. However, age- and sex-related differences in population-based normal values should also be considered when assessing QoL [39]. The influence of different types of urinary diversion on QoL in patients after RC remains a topic of debate. Both the study by Kretschmer et al. and the systematic review by Ghosh et al. showed better global health-related QoL in patients with an INB compared with patients with other types of urinary diversion [19, 20]. In contrast, the studies from Philip et al. as well as Hara et al., and the reviews from Ali et al., as well as Gerharz et al., have found no differences in global QoL between IC and INB patients [22-25]. According to Singer et al., patients report low QoL in both functional and symptom scales in the early postoperative period [40]. Functional scales take up to 12 months to recover, while symptom scales may recover within the first 6 months after surgery [41]. However, the summary score of the QLQ-C30, and not scores of single scales, appear to be a valid predictor for overall survival [42]. Furthermore, it should be kept in mind that certain aspects of quality of life may be affected by postoperative complications, which are significantly higher in our cohort than previously published [43, 44].

In a short and recent period from April 2018 to December 2019, we prospectively included 842 patients (IC *n* = 447, NB *n* = 395) from 135 different hospitals in Germany after RC and IC or NB creation in our study. Patients receiving an IC were significantly older (median 73 vs 64 years) and more often suffered from cardiovascular diseases and diabetes mellitus. Advanced tumor stage and lymph node metastases were significantly more often found in patients with an IC. Global health-related QoL improved steadily during follow-up, showed no significant differences between the two types of urinary diversion, and is comparable to the general population 12 months after surgery. However, patients undergoing adjuvant chemotherapy report significantly worse QoL. As diminished QoL of life has been shown to predict survival in several tumor entities, patients undergoing adjuvant treatment should be monitored closely [29-33]. Patients with an INB reported better physical function but suffered more from diarrhea and financial worries, while patients with an IC reported reduced satisfaction with their body image, increased worries about the future, and suffering more from constipation. Surprisingly, 12 months after surgery, QoL is comparable to the general population (64.6 vs 65.3). A significant difference when comparing QoL in patients with an IC and with an INB was not detected at any point in this study. Financial difficulties due to early retirement or reduced working hours are more common in younger patients. Job and financial insecurities are associated with an increased risk of depression [40]. If present, interventional steps should be taken as early as possible [45]. Fortunately, 80.5% of employed patients from this cohort returned to work 12 months after surgery.

An improved physical function in patients with an INB may be sufficiently explained by their younger median age. Meanwhile, patients with an IC report a significantly worse role function one year after surgery. Tyson et al. already reported, that patients with an IC suffer more from constipation, while patients with an INB report diarrhea significantly more often [46]. These findings were confirmed in our study. However, a “response shift” with a subsequent adaption may explain why these symptoms do not negatively influence QoL [47]. As already mentioned, QoL after RC in this cohort does not deviate significantly from the general German population. Nonetheless, role function, social function, and emotional function are worse in patients after RC. Generic questionnaires may not detect cancer-specific issues sufficiently. An analysis of QoL in cancer patients should always consider certain sub-scales, that may not affect the healthy percentage of the population.

Up to 89% of cancer patients criticized insufficient psychosocial care during therapy [48]. In our study, psychosocial distress increased significantly after discharge from IR. One year after surgery, 43.1% of patients still suffered from high psychosocial distress. Multivariate regression analysis identified an age ≤ 59 years and lymph node metastases as independent predictors for high psychosocial distress. According to several studies, women suffer from higher psychosocial distress during cancer therapy than men [5, 49, 50]. High psychosocial distress in female patients may have influenced our results. However, due to the majority of patients in our study being men, a significant influence on overall psychosocial distress does not seem likely and was consequently not investigated further.

Overall, QoL improves continuously after RC. However, high psychosocial distress persists in a substantial number of patients. Our data immediately identify patients at risk for high psychosocial distress. Psychosocial care of patients after discharge from IR was not recorded in our study. Results related to QoL should be interpreted with caution, as IC and INB patients are significantly heterogeneous in terms of baseline characteristics. The presence of cardiovascular comorbidities was identified as an independent predictor of worse health-related QoL in patients after RC [26]. The choice of the type of urinary diversion is a shared decision between the patient and the surgeon, especially considering age and comorbidity. Therefore, it is extremely unlikely that a prospective randomized trial comparing IC and NB will ever be conducted.

When advising patients concerning RC and urinary diversion, the impacts of QoL and influencing factors for psychological well-being should be considered. The offer to participate in support groups should be extended to all patients.

## Conclusion

Overall, quality of life improves continuously after RC, independent of the type of urinary diversion. However, a substantial number of patients suffer from psychosocial problems, which is why patients require further psychosocial care after discharge from the IR. To prevent chronic psychological disorders, easily accessible opportunities are needed to provide and encourage access to trained psycho-oncologists during aftercare in patients undergoing RC.

## Supplementary Information

Below is the link to the electronic supplementary material.

Supplementary file1 (DOCX 21 KB)

## Data Availability

Data are not publicly available but can be made available by the corresponding author upon reasonable request.
