# Supplementary material for: Psychosocial distress and quality of life in patients after radical cystectomy – one year follow-up in 842 German patients
Source: J Cancer Surviv. 2023 May 10;18(5):1600–7. doi: 10.1007/s11764-023-01400-6 (PMC11424671; doi:10.1007/s11764-023-01400-6)
Supplement: Supplementary file 1 — Supplementary file1 (DOCX 21 KB) [file 11764_2023_1400_MOESM1_ESM.docx]

**Table 2:** QLQ-C30 domains after inpatient rehabilitation following radical cystectomy

| **Variable** | **Total**  mean (SD) | **Conduit**  mean (SD) | **Neobladder**  mean (SD) | **p*** |
| --- | --- | --- | --- | --- |
| **Functional scales** |  |  |  |  |
| Global health status /  quality of life |  |  |  |  |
| T1 | 55.8 (19.7) | 55.6 (19.3) | 56.0 (20.1) | 0.872 |
| p** | <0.001 | <0.001 | <0.001 |  |
| T2 | 62.9 (21.2) | 61.9 (21.1) | 64.0 (21.3) | 0.292 |
| p*** | 0.034 | 0.614 | 0.012 |  |
| T3 | 64.6 (21.9) | 63.4 (21.9) | 65.8 (21.9) | 0.183 |
|  |  |  |  |  |
| Physical functioning |  |  |  |  |
| T1 | 64.3 (21.2) | 61.0 (22.0) | 68.0 (19.7) | < 0.001 |
| p** | <0.001 | <0.001 | <0.001 |  |
| T2 | 73.5 (22.5) | 68.9 (23.5) | 78.4 (20.4) | < 0.001 |
| p*** | 0.174 | 0.339 | 0.004 |  |
| T3 | 74.8 (22.7) | 69.2 (22.7) | 80.5 (21.2) | < 0.001 |
|  |  |  |  |  |
| Role functioning |  |  |  |  |
| T1 | 48.5 (31.7) | 48.1 (31.1) | 49.0 (32.4) | 0.674 |
| p** | <0.001 | <0.001 | <0.001 |  |
| T2 | 58.1 (30.8) | 55.7 (31.6) | 60.7 (29.7) | 0.051 |
| p*** | 0.036 | 0.738 | 0.007 |  |
| T3 | 61.7 (29.9) | 57.3 (29.3) | 66.1 (29.9) | < 0.001 |
|  |  |  |  |  |
| Emotional functioning |  |  |  |  |
| T1 | 70.0 (26.7) | 67.4 (27.5) | 72.8 (25.6) | 0.006 |
| p** | <0.001 | 0.007 | <0.001 |  |
| T2 | 66.1 (28.1) | 65.5 (29.0) | 66.7 (27.1) | 0.785 |
| p*** | 0.092 | 0.652 | 0.003 |  |
| T3 | 67.4 (27.7) | 65.8 (28.6) | 69.0 (26.7) | 0.202 |
|  |  |  |  |  |
| Cognitive functioning |  |  |  |  |
| T1 | 81.7 (23.4) | 81.1 (24.1) | 82.3 (22.6) | 0.621 |
| p** | 0.002 | 0.007 | 0.107 |  |
| T2 | 79.4 (25.0) | 78.2 (26.7) | 80.7 (23.1) | 0.514 |
| p*** | 0.747 | 0.791 | 0.491 |  |
| T3 | 80.0 (23.7) | 78.6 (24.7) | 81.4 (22.6) | 0.201 |
|  |  |  |  |  |
| Social functioning |  |  |  |  |
| T1 | 62.4 (31.1) | 63.8 (31.1) | 60.8 (31.0) | 0.134 |
| p** | 0.008 | 0.006 | 0.307 |  |
| T2 | 61.1 (31.3) | 61.3 (32.1) | 60.9 (30.5) | 0.669 |
| p*** | 0.001 | 0.201 | 0.001 |  |
| T3 | 64.4 (30.3) | 64.6 (30.3) | 64.2 (30.5) | 0.842 |
|  |  |  |  |  |
| **Symptoms scales** |  |  |  |  |
| Fatigue |  |  |  |  |
| T1 | 45.1 (26.3) | 47.9 (26.9) | 42.0 (25.3) | 0.002 |
| p** | 0.002 | 0.003 | 0.120 |  |
| T2 | 40.7 (27.9) | 42.8 (29.1) | 38.6 (26.4) | 0.132 |
| p*** |  | 0.405 | 0.008 |  |
| T3 |  | 41.9 (29.0) | 35.8 (27.1) | 0.010 |
|  |  |  |  |  |
| Nausea and vomiting |  |  |  |  |
| T1 | 6.0 (16.1) | 7.3 (18.2) | 4.6 (13.3) | 0.037 |
| p** | 0.098 | 0.339 | 0.169 |  |
| T2 | 6.4 (16.3) | 7.3 (17.8) | 5.5 (14.6) | 0.205 |
| p*** | 0.202 | 0.314 | 0.396 |  |
| T3 | 38.9 (28.2) | 7.3 (19.2) | 5.1 (13.7) | 0.515 |
|  |  |  |  |  |
| Pain |  |  |  |  |
| T1 | 23.0 (27.6) | 24.1 (29.0) | 21.8 (25.9) | 0.584 |
| p** | 0.406 | 0.556 | 0.554 |  |
| T2 | 20.5 (27.6) | 20.7 (28.8) | 20.2 (26.4) | 0.689 |
| p*** | 0.744 | 0.123 | 0.026 |  |
| T3 | 20.7 (27.5) | 22.1 (28.9) | 19.3 (26.0) | 0.456 |
|  |  |  |  |  |
| **Single items** |  |  |  |  |
| Dyspnoea |  |  |  |  |
| T1 | 25.5 (30.5) | 30.4 (33.0) | 20.2 (26.5) | < 0.001 |
| p** | <0.001 | 0.183 | <0.001 |  |
| T2 | 30.0 (31.8) | 32.1 (33.4) | 27.9 (29.9) | 0.177 |
| p*** | 0.556 | 0.011 | 0.092 |  |
| T3 | 30.6 (32.1) | 34.2 (33.3) | 27.1 (30.4) | 0.012 |
|  |  |  |  |  |
| Insomnia |  |  |  |  |
| T1 | 35.9 (34.4) | 37.5 (34.8) | 34.2 (33.9) | 0.191 |
| p** | 0.463 | 0.315 | 0.983 |  |
| T2 | 35.6 (34.8) | 37.9 (35.4) | 33.2 (33.9) | 0.092 |
| p*** | 0.179 | 0.982 | 0.057 |  |
| T3 | 34.9 (34.5) | 38.3 (34.8) | 31.6 (33.9) | 0.016 |
|  |  |  |  |  |
| Appetite loss |  |  |  |  |
| T1 | 24.6 (31.8) | 27.3 (33.9) | 21.6 (28.9) | 0.049 |
| p** | <0.001 | <0.001 | <0.001 |  |
| T2 | 15.7 (24.4) | 17.7 (29.1) | 13.5 (25.4) | 0.048 |
| p*** | 0.934 | 0.209 | 0.121 |  |
| T3 | 14.1 (25.4) | 16.9 (27.5) | 11.2 (22.7) | 0.005 |
|  |  |  |  |  |
| Constipation |  |  |  |  |
| T1 | 25.4 (32.4) | 30.5 (34.4) | 19.8 (29.0) | < 0.001 |
| p** | 0.243 | 0.929 | 0.044 |  |
| T2 | 22.1 (29.9) | 27.9 (32.8) | 15.8 (25.0) | < 0.001 |
| p*** | 0.921 | 0.756 | 0.812 |  |
| T3 | 21.8 (29.9) | 26.7 (33.0) | 16.8 (25.7) | < 0.001 |
|  |  |  |  |  |
| Diarrhoea |  |  |  |  |
| T1 | 16.6 (27.5) | 15.1 (26.8) | 18.2 (28.1) | 0.064 |
| p** | <0.001 | 0.610 | <0.001 |  |
| T2 | 21.0 (28.8) | 15.2 (25.6) | 27.2 (30.7) | < 0.001 |
| p*** | 0.298 | 0.377 | 0.538 |  |
| T3 | 21.0 (28.4) | 13.5 (23.9) | 28.5 (30.5) | < 0.001 |
|  |  |  |  |  |
| Financial difficulties |  |  |  |  |
| T1 | 20.7 (30.1) | 17.3 (28.3) | 24.5 (31.6) | 0.001 |
| p** | <0.001 | 0.113 | 0.001 |  |
| T2 | 23.0 (32.3) | 19.1 (29.7) | 27.1 (34.4) | 0.002 |
| p*** | 0.042 | 0.262 | 0.082 |  |
| T3 | 19.7 (30.5) | 15.9 (27.3) | 23.6 (33.2) | 0.006 |

**Abbrevations:**

T1 = end of inpatient rehabilitation

T2 = 6 months after discharge from inpatient rehabilitation

T3 = 12 months after discharge from inpatient rehabilitation

SD = standard deviation

*****Mann-Whitney-U test (conduit vs. neobladder)

**Wilcoxon-test (T1 vs. T2)

***Wilcoxon-test (T2 vs. T3)
